# Designing an e-learning tool to support health practitioners caring for patients taking multiple medications

**DOI:** 10.12688/hrbopenres.13110.2

**Published:** 2021-04-06

**Authors:** Michelle Hanlon, Michael Hogan, Hannah Durand, Monika Pilch, Owen Harney, Gerard Molloy, Andrew W. Murphy

**Affiliations:** 1 School of Psychology, NUI Galway, Galway, H91 TK33, Ireland; 2 Centre for Health Policy and Management, School of Medicine, Trinity College Dublin, Dublin 2, Ireland; 3 The Ryan Institute, NUI Galway, Galway, H91 R8EC, Ireland; 4 HRB Primary Care Clinical Trials Network Ireland, NUI Galway, Galway, Ireland; and College of Medicine Nursing & Health Sciences, NUI Galway, Galway, Ireland

**Keywords:** Multimorbidity, Polypharmacy, Adherence, General Practice, PPI, Collective Intelligence Design, E-learning tool

## Abstract

**Background:** Population ageing and improvements in healthcare mean the number of people living with two or more chronic conditions, or ‘multimorbidity’, is rapidly increasing. This presents a challenge to current disease-specific care delivery models. Adherence to prescribed medications appears particularly challenging for individuals living with multimorbidity, given the often-complex drug regimens required to treat multiple conditions. Poor adherence is associated with increased mortality, as well as wasted healthcare resources. Supporting medication adherence is a key priority for general practitioners (GPs) and practice nurses as they are responsible for much of the disease counselling and medication prescribing associated with chronic illnesses. Despite this, practical resources and training for health practitioners on how to promote adherence in practice is currently lacking. Informed by the principles of patient and public involvement (PPI), the aim of this research was to develop a patient informed e-learning resource to help GPs and nurses support medication adherence.

**Method:** Utilising collective intelligence (CI) and scenario-based design (SBD) methodology, input was gathered from key stakeholders in medication adherence to gain insights into barriers to supporting people with multimorbidity who are receiving polypharmacy, strategies for overcoming these barriers, and user needs and requirements to inform the design of the e-learning tool.

**Results: **In total, 67 barriers to supporting people who are taking multiple medications were identified across 8 barrier categories. 162 options for overcoming the identified barriers were then generated. This data was used in the design of a flexible e-learning tool for continuous professional development, that has been integrated into general practice and clinical education programmes as a supportive tool.

**Conclusions: **Using CI and SBD methodology was an effective way of facilitating collaboration, idea-generation, and the co-creation of design solutions amongst a diverse group of stakeholders. This approach could be usefully applied to address other complex healthcare-related challenges.

## Introduction

Healthcare utilisation and cost in both primary and secondary care is significantly increased with multimorbidity (i.e., co-occurrence of two or more chronic conditions). Each additional chronic condition leads to near-exponential increases in both service usage and financial costs ( McPhail, 2016). This effect on primary care consultations, hospital outpatient visits and admissions and total healthcare costs occurs independently of age, gender and socioeconomic status ( Glynn *et al.*, 2011). The cost of living with multimorbidity is also high for the patient. Living with multiple conditions has debilitating physical, psychological, social, and financial consequences and is associated with impaired functional capacity, reduced quality of life and increased rates of psychological distress ( Fortin *et al.*, 2004; Ryan *et al.*, 2015). The treatment burden individuals with multimorbidity experience is also significantly high as each condition can require different treatments, lifestyle adjustments, specialist care, and medications.

In order to increase the quality of care being delivered to patients and reduce spiralling healthcare costs, there is a need to support people to self-manage their chronic health conditions ( HIQA, 2015). Supporting people to self-manage their health through education and supportive interventions can increase skills and confidence, enhance self-efficacy, and improve day-to-day quality of life. It can also lead to improvements in clinical outcomes, reduce healthcare utilisation, and decrease hospitalisation ( HSE, 2015). For the majority of patients, self-management shows significant benefits when there is increased support from healthcare professionals ( Grady & Gough, 2014). Self-management support may include such components as case management, frequent follow-ups and patient education ( HSE, 2017). Examples of self-management education may include providing information about the patient’s condition(s), advice on exercise and nutrition, and guidance on appropriate use of medications.

Medication adherence can be a particularly challenging aspect of living with multimorbidity due to high treatment burden. The term ‘adherence’, as opposed to ‘compliance’, is critical, as it highlights the active role of the patient in their treatment ( Vrijens *et al.*, 2012). If patients do not take medications as prescribed, then they are unlikely to receive the full benefits of treatments that we know work. Poor adherence can also lead to unnecessary suffering and wasted resources ( Cutler *et al.*, 2018; van Boven *et al.*, 2014; Walsh *et al.*, 2019); despite this, patients are often reluctant to tell their doctor or nurse that they do not take their medicines as prescribed. If a patient's medication taking behaviour is not understood, therapy may then be needlessly escalated ( Brown & Sinsky, 2018). Escalating therapy when non-adherence is not identified can cause potential harm to the patient, create unnecessary work for the practice, and result in increased costs to the patient and healthcare system. However, the reasons behind medication non-adherence are complex, and can go beyond a lack of information, forgetfulness, or even access to medication itself ( Kardas *et al.*, 2013). Understanding the complexity of factors associated with non-adherence is therefore key to addressing the issue, and any tool designed to support patient adherence should be designed with the voice of the patient in mind. Thus, informed by the principles of patient and public involvement, the aim of the current project was to create an e-learning resource that would use evidence-based approaches to help healthcare professionals to support long-term medication taking in multimorbidity. As general practitioners deliver continuous care to patients and are responsible for much of their medication prescribing, the resource that was created, aminuteforadherence.ie, was designed to be an interactive and easily accessible resource that could be integrated in to general practice and clinical education programmes as a supportive tool. Such a training resource was lacking as summaries of treatment adherence have generally been more research than clinical management orientated ( Sinclair *et al.*, 2016). The term ‘collective intelligence’ (CI) is used to describe intelligence, or knowledge, that emerges as a result of a group of people working together to come up with solutions to a problem. The CI approach carefully delineates content and process roles, assigning to experts the responsibility for contributing ideas, and to the workshop facilitator responsibility for choosing and implementing selected methodologies for generating, clarifying, structuring, interpreting, and amending ideas. Emphasis is given to balancing behavioural and technical demands of group work ( Broome & Chen, 1992) while honouring design laws concerning variety, parsimony, and saliency. There must be enough variety in options to cover all needs, but possible paths and solutions must exist in harmony to avoid creating confusion or disrupting problem resolution ( Ashby, 1958; Boulding, 1966; Miller, 1956). Using CI can help to support high quality interdisciplinary work as it includes a set of methods and tools and a facilitated thought and action mapping process that helps groups to develop outcomes that integrate contributions from individuals with diverse views, backgrounds, and perspectives ( Hogan *et al.*, 2014; Warfield & Cardenas, 1994). CI has been applied in many different situations to accomplish many different goals, including mediating peacebuilding in protracted conflicts ( Broome, 2006), developing a national well-being measurement framework ( Hogan *et al.*, 2015), mobilising communities across Europe in response to marine sustainability challenges ( Domegan *et al.*, 2016), and understanding and overcoming barriers to the design of personalised nutrition products and services for older adults ( Hogan *et al.*, 2017).

The current research project brought together general practitioners, practice nurses, pharmacists, medical educators, psychologists, learning technologists, as well as members of the public living with multimorbidity and receiving polypharmacy. One of the primary advantages of using CI was the facilitation of communication between potential e-learning tool users in relation to usage possibilities and the challenges that may arise for different stakeholders.

## Methods

### Participants

A total of 16 stakeholders with lived experience of multimorbidity and taking polypharmacy, and/or general practice, nursing, psychology, education, and pharmacy backgrounds participated in the collective intelligence design work. The workshop took place on April 6 2018 in the School of Psychology in NUI Galway. A purposive sampling strategy was utilised to ensure that a diversity of healthcare professionals, researchers and patient groups (e.g., young and older adults) were represented in the sample. Guidance on patient involvement was sought from PPI specialists within NUI Galway, who facilitated recruitment of people living with multimorbidity. Healthcare professionals and researchers were recruited via existing professional networks. A summary of participant backgrounds can be found in Table 1.

**Table 1.** Collective Intelligence Workshop Participants.

Participant Number	Stakeholder Representation
1	General Practitioner
2	Lecturer in Psychology
3	General Practitioner & Lecturer in General Practice
4	Researcher, specialising in treatment adherence
5	Researcher, HRB Primary Clinical Trial Network and PPI Ignite
6	Research Fellow, School of Psychology
7	General Practice Nurse
8	PhD Candidate, specialising in treatment adherence
9	PhD Candidate, specialising in treatment adherence
10	Senior Lecturer in Education
11	PPI Ignite @ NUI Galway programme manager
12	Community Pharmacist
13	Patient Representative
14	Patient Representative
15	Patient Representative
16	Patient Representative

### Collective intelligence

**Figure 1.**
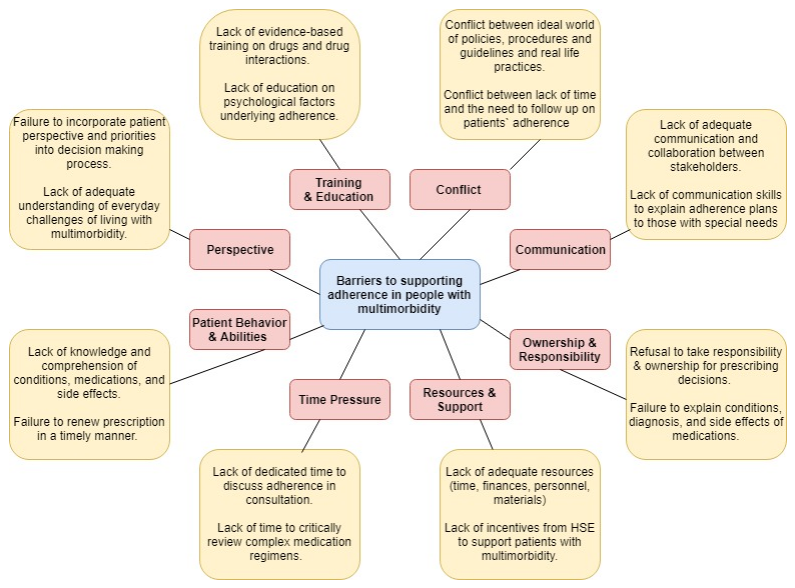
Sampling of barriers to medication adherence.

Collective intelligence methodology was used for this research as, while it is more time consuming than other qualitative methods in the design phase, it provides a structured and systematic way of solving a complex problem and utilises a carefully selected set of methodologies that can be matched to the phase of group interaction and the requirements of the situation. For this project, the CI methodology that was used included idea-generation and idea categorisation, combined with scenario-based design. The first stage of the CI process involved systematic analysis of barriers to supporting people with multimorbidity, which helped guard against the use of rigid thinking patterns in relation to the e-learning system and the tendency toward solution-first problem solving and design thinking ( Rosson & Carroll, 2002). Stakeholders with expertise related to the problem context were contacted in advance of the session by email, with a request to generate a set of barriers in response to the following trigger question: “ *What barriers do healthcare practitioners face in supporting people with multimorbidity who are taking multiple medications?*". By email submission, experts identified 67 barriers. These responses were subsequently reviewed by the workshop facilitation team and categorised using the Paired Comparison Method ( Warfield & Cardenas, 1994). The paired comparison method provides a simple way of summarizing a group of individuals opinions, attitudes or beliefs about a topic in a systematic and objective manner. Responses to a question are clustered into categories, based on similarity, so that the group can see an overview of the issues within each category and work out the most important problems to solve. In this case, eight distinct categories were identified, providing a focus for initial discussions at the CI workshop. Each category highlighted distinct issues or barriers to medication adherence: Training and Education; Conflict; Communication; Ownership and Responsibility; Time Pressure; Resources and Support; Patient Behaviour and Abilities; and Perspective. A sampling of categorisation of barriers can be seen in Figure 1 and a complete list of barriers is presented in Tables 2–Table 9.

**Table 2.** Barriers and options: Training and Education.

Barrier: Training and Education
**Inadequate training in supporting people with adherence.**
**Inadequate knowledge of behaviour-change techniques.**
**Lack of in-depth knowledge of drugs and drug interactions.**
**Inappropriate structures within EHR, lost opportunity in terms of Computerised Decision Support Systems.**
**Lack of evidence to support decision-making.**
**Lack of psychological education to understand patients’ personality type and its influence on attempts to** **promote medication adherence.**
Options for overcoming barriers
Set up open access for health care professionals (HCP) to new evidence on needs/interactions – available online (e.g., on a monthly basis).
Promote software vendors ** + HSE to develop an app with access to real time patient medications.
Improve current drug interactions software.
Demand better health record options to become more 21 ^st^ century.
Discuss ways of alleviating side effects of medications.
Encourage a process to consider side effects profile during consultations.
Create an education module on the psychology of adherence (behaviour change techniques that are effective; intentional vs. unintentional non-adherence).
Improve GP access to adherence and side effects literature (e.g., quick summaries).
Organise accessible ways for sharing information.
Encourage information on side effects to be weighed against the effects of taking drugs.
Point patients in the direction of information.
Conduct education courses.
Use GP practice waiting rooms screens to share information on why adherence is important.
Identify a reliable and evidence-based website to refer patients to regarding on side effects, importance of adhering, etc.
Encourage patients to understand they should return to discuss side effects before they decide to stop taking meds.
Create electronic learning resources for GPs/practice nurses and separate for patients.
Encourage the uptake of an app with the patient’s medication list.
Change how information is shared between HC sites – GP/Hospitals/Pharmacy/ Out of hours.
Create database of interventions with proven evidence of efficacy.
Build pathways for information sharing between patients/GP/hospitals/pharmacy.
Create a learning tool for GPs which fulfil their IMC audit criteria.

**Table 3.** Barriers and options: Time Pressure.

Barrier: Time Pressure
**Lack of time to critically review complex medication regimen and take holistic patient overview.**
**Lack of time to undertake medicine reviews.**
**Time constraints make it difficult for doctors to have a detailed discussion with patients about** **issues they are having with adhering to medication.**
**Failure to designate time to medication adherence. Patient consults focus on gathering** **information and diagnosing. Neither doctor nor patient have full focus on adherence.**
**Lack of time in the consultation.**
**Lack of time to research into best combinations of medication use -prescribing based on what** **you know rather than what is the best available.**
**Patients often do not have enough time to speak about their problems.**
**Doctors do not have enough time to explain to patients what exactly the medication is for, how it** **is to be taken, how long it is to be taken for, the consequences of not taking it.**
Options for overcoming barriers
Encourage patients to refer to pharmacy re adherence.
Promote adherence audits in practices categorising patients as adherent/high, etc.
Design a patient friendly adherence education tool that GPs and pharmacists could refer patients to.
Encourage GPs to designate 1 minute of the consultation to adherence.
As part of audit, explore patients` prescriptions & level of risk of non-adherence; prioritise conversations with these patients.
Conduct focus groups with patients – how would they like their GP to bring up adherence? Would they prefer a pharmacist?
Demand more practice nurses.
Establish how to audit and efficiently measure adherence in the consultation.
Link in with patient groups and ask them to emphasise adherence.
Promote that pharmacist ask about adherence.
Develop a system so that GPs and pharmacists can identify high risk patients.
Establish best practice in medicine reviews in learning tool.
Establish that non-adherence is a real problem (for the health system; for the individual patient).
Develop a support for patients to pro - actively organise themselves.
Medication review audit – flag high risk patients (asymptomatic, high risk conditions, personal circumstance, number of medications).
Promote a minute for adherence within the GP consultation = 22 million minutes a year = 15,277days.
Encourage patients to propose a checklist of questions for the GP.
Encourage practice nurses to also address adherence issues with individual patients.
Promote engagement with patient organisation as patient adherence.
Increase awareness on importance of adherence.
Create an education resource for GPs around the importance and impact of adherence.
Apps with flags for “priority” patients to ensure conversations happen.
Have patients fill out questionnaires about medication adherence in the waiting rooms.
Waiting room time: Use screens in HC practice to inform about importance of adherence (ted talks, pod casts, posters, leaflets, iPads).

**Table 4.** Barriers and options: Conflict.

Barrier: Conflict
**Conflict between ideal world of guidelines for individual conditions and daily practice of patients with** **multiple problems.**
**Conflict between evidence-based guidelines, medicines prescribed and patient preferences.**
**Conflict between patients and doctors on adherence may impact on patients actually not attending** **practices for regular review.**
**Conflict between the clinician’s medical decision and the patients autonomy.**
**Conflict between being time poor and caring about follow up and outcomes.**
**Conflict between maintaining the relationship with the patient and being overly-paternalistic.**
**Conflict between respecting the patient’s autonomy and supporting them to change their behaviour.**
Options for overcoming barriers
Set – up an app where the patient can put in daily accounts of medication use, to enhance knowledge of adherence for both GP and patient.
Encourage the patient to change their behaviour but do not force/judge/micro manage as is important to respect patient autonomy (can`t chase everything).
Create an online system offering up – to – date advice, information, education about multi-morbidity and multiple medications.
Create an open access and non-judgemental atmosphere in the GP`s room between the patient and doctor to allow an open and honest conversation.
Encourage patients to self-monitor adherence for a period of time so that adherence issues can be identified objectively.
When new medications are prescribed, encourage the patient to make a follow up appointment so you can discuss adherence/side effects properly. Not a month later. Follow up apps are very important.
Develop an accreditation service with certified knowledge to encourage self-management instead of google false knowledge.
Dedicate time within the consultation to address medication taking in an open and honest way.
Establish a patient and GP association.
Develop an online GP service similar to online/email counselling so time is less of a worry.
Create clusters of GPs and patients, based around specific adherence and medication combinations and multimorbidity.
Promote an open discussion at the start of the session, where each person takes time to listen to another with empathy.
Conflict between adherence cannot cause too much of an issue, doctors must take the patient at face value and not assume, or the relationship may become damaged Develop a new plan if needed.
Promote and raise awareness of multi-morbidity. Education.
Discuss patient priorities in a non-judgemental way, e.g. if patient prioritises pain management over BP control, discuss implications of this.
Education to counter misinformation/erroneous advice.

**Table 5.** Barriers and options: Communication.

Barrier: Communication
**Lack of communication and collaboration between those caring for the patient, e.g. between the different** **clinics and consultants they are attending.**
**Inability to explain coherent adherence plans to some patients, including patients with dementia.**
**Lack of in-depth follow up with patients following prescribing.**
**Miscommunication between health care professionals and their patients.**
**Patients not being able to communicate which of their conditions is exacerbating their physical or mental state.**
**Inability to maintain appropriate and accurate communication with other health care professionals, e.g.** **hospital staff, pharmacists, etc.**
**Inadequate conversation about side-effects and medication.**
**Healthcare professionals often lapse in to ‘medical jargon’ while issuing instructions and do not check if the** **patients understand.**
**Varying messages from different healthcare professionals.**
Options for overcoming barriers
Plan to meet the patient after all relevant information from other healthcare providers, e.g. consultants, is required?
Involve pharmacists in all medication interactions, the GP cannot be expected to know everything about drugs – nor the pharmacist.
An annual/6 monthly review of medication-whether required or not.
Holistic approach in that communication from different consultants being combined – not all left individually.
Demand change in communication process, using audit recording of consultant advice.
Explain purposes of medication (simply) and benefits.
Conduct a medication and adherence review annually.
Promote discussion around adherence difficulties in consultations.
6 months system: review of medication.
Patient questionnaire.
Promote support call from practice nurse, pharmacy (side effects/adverse events, working/networking).
Reporting function to HPRA.
Medication support/knowledge workshop.
Text reminders, medication review.
Encourage doctors to interact with patients – time factors. Empathy concern for patient to be palpable. **
Encourage patients to speak about how they manage their medicines at home.
Train GPs to elicit from the patient what their main concern is at each visit.
Encourage patients to be accompanied to GP to learn & absorb & question what is communicated.
GP to have simple format or method ** of what to look for.
5Ws app: Who/what/where/why/when; SMART: how much/often/review/repeat (specific).
Communication cycle ; Engagement Tools/techniques.
Plan for individual needs, e.g. cognitive capacity of individual.
Encourage patients to bring family/other support to consultations to facilitate communication.
Make GPs aware of the delay in consultant letters.
Set up an adherence consultation that focuses on managing all patient medications.
Patient self-reporting function.
Medication counselling.
Refill Data.
Include (in the e – learning tool) a case study of how to explain coherent adherence plan – using a “bad” example, discuss it, and then give same “good” example.

**Table 6.** Barriers and options: Patient Behaviour and Abilities.

Barrier: Patient Behaviour and Abilities
**Some patients take the medications home but do not use them.**
**Patients’ refusal to take or not take medications as prescribed due to lack of education, how it feels,** **effects on lifestyle, etc.**
**Inadequate knowledge in that the patients will describe that they are taking ‘the blue pill’ and not** **necessarily understand what it is for.**
**Not renewing the prescription in a timely manner. This means the patient has missed doses or is out** **of them for a while.**
**Patients inability to identify difference between generic medicines and alternatives.**
**Patients may not understand the advice given or instructions on accompanying literature.**
**Patient in denial of condition.**
**Lack of honesty by the patient. Some patients do not take for example their diuretics and don’t tell** **the doctor/nurse so then the dose is increased as it ‘isn’t working’.**
**Confusion over times medicines are meant to taken.**
**Inability to understand that medication must be adhered to for optimal result.**
**Patient may not understand the importance of taking medication at the times and strengths** **prescribed.**
Options for overcoming barriers
Encourage active relationship with pharmacist and establish history with them.
Develop an experiential learning component for GP training that involves seeing the day of someone with multi- morbidity & polypharmacy.
Encourage GPs to emphasise importance of adhering every time/encounter with patient.
Promoting sharing cost of medication with all patients.
Consider selective removal of drugs for a set amount of time + ask: Are you feeling the same, better or worse? Make a decision then.
Promote action planning to take medications at specific times that make it easy.
Clarification and acknowledgment that a patient understands dosage and timing.
Emphasise to healthcare professionals that patients forget/don`t understand – so repeat, repeat and repeat information.
Develop more patient cantered drug information that manages potential fear around taking meds + positive information.
Create templates to help different type of patients to self – manage adherence.
Doctor/pharmacist communicate the “whys” of taking medication.
Establish if any potential adherence solutions are suitable for a given individual, e.g. apps, blister packs not suitable for all.
Review medication & stick with originals not generics.
Create prompts for phone or other electronic reminders.
Make “Coping and Acceptance Skills” workshop available to those in denial.
Train healthcare professionals to communicate the basics of coping + acceptance skills.
Establish patient priorities for medications and related symptom/disease management.
Tools/Tips to take medication.
Develop Action plans for adherence –leave medications on breakfast table or beside the kettle so will remember to take tablets in the morning.
Encourage to use alarm as a reminder.
Medication counselling.
Continuous relationship with pharmacy/GP- it is very important to attend the same one each time.
Coping skills.
Education on medicine management for patients.
Waiting room – research projects – get them active while waiting – surveys.
Educate while waiting – you as GP and them as patients.

**Table 7.** Barriers and options: Ownership and Responsibility.

Barrier: Ownership and Responsibility
**Refusal by the physician to accept responsibility for non-adherence, for example, due to fear of potential side effects.**
**Insufficient exchange of information between patient and prescriber – e.g. what side effects to expect from** **medications, and what should and should not be tolerated.**
**Lack of open communication with patients and limited ownership of all prescribing decisions, as some drugs are** **prescribed by different consultants.**
**Failure to explain condition to patient – very often diagnosis not explained to patient by healthcare professional.**
**Lack of reporting of side effects or adverse events by patients – they just stop taking the medication.**
**Patients may use denial as a way to deal with their diagnosis – and this may involve refusal to accept changes or the** **extent to which lifestyle must be modified.**
**The person taking the pill combination could be unwilling to articulate the extent of their incomprehension, perhaps** **due to feelings of inadequacy or they may also be unaware of this incomprehension.**
Options for overcoming barriers
Set up a system of continued communication between GP/Hospital; Patient Personal Card.
More co-ordination between the patients, GPs and clinics they are attending.
Specific educational booklet on specific condition.
Patient encouraged to read/watch/listen to educational materials provided and to and ask ** relevant questions at their next visit.
Ideal world, holistic education (not financially viable / time and resources).
Patient responsibility.
Explain, explain, explain; Promote explanation.
Community pharmacy.
Create a unique patient file between 1 ^st^ and 2 ^nd^ degree care; Paper based; Patient headings/ meds for each outpatient appointment; Unique 1 and 2 degree care IT patient file/smartcard.
Explain what side effects may occur- when patient feels a reaction they may be more likely to tolerate it or bring it up with GP at next appointment.
Patient needs to take more responsibility, i.e. keep record of medications, update regularly to discuss with GP.
Do not assume the patient`s understanding, explain regardless.
Set – up community pharmacy roles within Primary Care teams.
Explanation of meds/ conditions.
Working with those who have adherence issues.

**Table 8.** Barriers and options: Resources and Support.

Barrier: Resources and Support
**Inadequate support from the HSE.**
**Inadequate resources, particularly time and personnel resources.**
**Inability of patients to pay for multiple medications, costs associated with attending multiple appointments.**
**Lack of staff/patient support material.**
**Lack of incentives – health care professionals are not incentivised to provide the level of time and support** **required to care for some patients.**
**Lack of timely, bespoke provision of healthcare to those with complex multimorbidity.**
**Lack of support for healthcare practitioners to support people with multimorbidity who are taking multiple** **medications.**
**Lack of access to relevant and timely healthcare.**
**Lack of time and resources impact on patient management and care.**
**Inadequate support and engagement with GP’s from the medicines management programme.**
Options for overcoming barriers
Create teams of GPs to support adherence and ameliorate lack of/limited resources and supports.
Leverage the risk to dedicate more funding to enhance adherence in order to reduce loss of finances due to medication waste.
Education resource to teach public prior to appointment.
Establish a way that more researchers can be involved in the work (academic support and vocational support).
Utilise expertise from other disciplines (e.g. psychology, behavioural science) within the practice.
Educate the public/patient about the cost of drug misuse. Do an advert. See where this money could be used elsewhere.
Transfer knowledge to GPs/patients, etc. Publish material and make them aware. Re-write materials in informal language (more explanation). Will allow for more support and informative material.
Create an informative system to promote staff/patient support material in just-in-time format.
Incentivise medication reviews, e.g. by fulfilling audit requirements.
Develop support materials for patients and GP staff that translate best practice clinical guidance and empirical research into lay language.
Create incentives for GPs to provide level of time and support required to care for multimorbidity patients, etc. CPD points.
Ethics issues here.

**Table 9.** Barriers and options: Perspective.

Barrier: Perspective
**Failure to incorporate patient perspectives and priorities into decision making.**
**Failure to have an open discussion about side effect that the person might be experiencing that might affect** **adherence.**
**Lack of understanding of everyday challenges of living with multimorbidity, which are exacerbated when on a low** **budget.**
**Limited understanding of patients’ beliefs and concerns about taking multiple medications over time.**
**Lack of perspective from health care professionals regarding the importance of their role in supporting patients.**
**Inability to establish or fully understand what matters most to the patient.**
**Failure to appreciate the emotional and psychological demands of multimorbidity.**
**Inadequate understanding of potential physical limitations and restriction for those with multimorbidity when** **accessing health care.**
**Fear of asking practitioners about medications.**
Options for overcoming barriers
Adherence: establish whether patients are actually fully compliant.
“How many times have you not taken your meds?”; “If so, why?” Reason – side effects.
Multiple meds for multiple conditions. Educate patients to understand conditions and to understand reasons for taking their specific medications.
Cost: evaluate if they have a medical card or not.
Lack of perspective due to insignificant time; Doctors may have already explained it several times.
Develop a “safe” place to allow discussions about non-adherence, side effects, dissatisfaction with therapy.
Organise the practice to highlight multimorbidity as a clinical entity with its own specific challenges.
Develop plans to help HCP understand their crucial role in multi-morbidity.
Promote patients to express their beliefs.
Adherence; How to do; Best practice.
PT involvement in care non-adherence plans.
Multi-morbidity as an entity, coping, adherence.
Participants assumed symptom HCP mixes.
Everyday challenges – juggling family, work, etc. on top of managing multiple conditions + their multiple appointments + medication schedule more coordination between healthcare providers and involve pharmacists to see if can ease medication burden, etc.
Inability to establish and fully understand about what matters most to patient. Develop more open discussion is needed.
Failure to appreciate emotional and psychological demands and more focus on the psychological impact of having multimorbid conditions + educating on supportive tools to manage living with multimorbidity and how emotional side can affect everything + impact adherence.
Knowing patients` beliefs, family situations.
Involving patients to ensure that it is THEIR plan that suits THEIR lifestyle (involving them in the process).
Couching the language in such a way to challenge people`s defence mechanisms.
Setting up a system where a patient had a say.

On the day of the workshop, the room was set up in such a way that the ‘problem field’ (i.e., the statements received and the categories into which they had been organised) were displayed around the room on poster boards. Participants were divided into four groups by the CI facilitators prior to the workshop, to make sure that a mix of stakeholder expertise was represented at each table. This ensured that a variety of perspectives would be attained during each of the exercises that would be given throughout the day. A short presentation providing an overview of the current position on multimorbidity and treatment adherence was delivered to provide context for the activities that would be completed throughout the day. Participants were advised that their feedback, ideas, and suggestions would be used to inform the design of an e-learning tool to support health practitioners caring for this group of patients. All participants gave permission for the facilitator to record the presentations that were going to take place throughout the day and for their written work to be collected after each exercise. The anonymised audio recordings are stored in a password-protected file on the lead researcher’s password-protected computer and the anonymised worksheets are stored in a locked drawer in a locked office in the University. In line with University policy, the data will be stored in this secure location, with access limited to the researchers on the project, for 10 years. Workshop participants then engaged in an analysis of the eight categories of barriers, with a view to generating options for overcoming barriers faced by healthcare practitioners in supporting people with multimorbidity who are prescribed multiple medications. In order to generate options in response to barriers, small working groups of participants (4–6 persons each) engaged in idea generation in response to assigned barrier categories. During this phase of the workshop, the ideawriting technique was used ( Wood & Roth, 1990). Each of the small groups was asked to generate options in writing and to use open dialogue to explore the meaning of ideas generated. Five steps were involved in idea writing: (a) a stimulus question was presented to participants; (b) each participant worked alone to silently generate ideas in writing; (c) written sheets of ideas were exchanged among all group members and individuals had the opportunity to add ideas as they read others’ papers; (d) unique ideas were discussed and clarified; and (e) each working group orally reported the ideas generated in a plenary session. This phase of work focused on the generation of options in response to barrier categories and allowed stakeholders to scope out a broad range of options in response to barriers before focusing attention on specific scenarios of usage for the proposed e-learning support tool.

The next phase involved using scenario-based design methods to generate specific user needs ( Rosson & Carroll, 2002). In advance of the CI session, the research team and facilitation team worked together to design a set of scenarios that could be used as inspiration for group idea generation during the workshop ( Table 10). Following guidelines provided by Rosson & Carroll (2002), design representations captured in the scenarios were concrete, flexible, generative and did not specify fixed solutions. As such, scenarios were designed to elicit constructive cognitive processes and the development of creative and bespoke solutions that are suitable for the specific context of usage in the scenario. In this way, a collaborative analysis and elaboration of needs is encouraged in the exchange between the design team and stakeholders. The scenarios that were used depicted a series of challenges faced by multiple actors (general practitioners, practice nurses, patients, carers, and pharmacists) in order to help workshop participants to orient themselves in each individual ‘actors’ problem space, so that they could imagine such a situation arising and consider what supports would be useful in each of these circumstances. Specifically, the aim was to prompt user needs in relation to: (1) Information and Knowledge; (2) Communication Needs; (3) Decision-Making Support; (4) Behavioural Support Needs; (5) Relational Needs; and (6) Other Needs. Participants were asked to consider the roles of the different actors in each scenario and to generate a list of needs for each actor and the reasons for these needs. These needs were subsequently discussed by sub-groups and all ideas were collated by the workshop facilitation team. The identification of user needs generated through this scenario-based design phase, informed by the earlier CI analysis of barriers, and generation of targeted options, provided a strong basis for further design work for the final resource that was created.

**Table 10.** Scenarios Used in the Workshop.

**A.**	GP George is interested in quickly accessing and reviewing information on medication adherence and behavioural supports designed for patients with multiple chronic conditions. As a busy general practitioner, George would like to know how to best communicate with this group of patients and involve them in a decision-making process, irrespective of time constraints. He is concerned about his patient Joe who is diagnosed with multiple chronic conditions but who is stressed about having to take multiple medications and is worried about their side effects. He wants to help his patients understand the importance of taking medication and support them in the process of adjusting to their new conditions and medication regimens.
**B.**	Caroline is a community nurse in a rural town and would like to be able to access relevant information and knowledge and decision- making tools while travelling around and visiting patients with multiple chronic conditions. She wants to access information on behavioural supports to help her patients remember to take their medication. She also would like to find tools that would facilitate two-way communication between patients and broader care network (e.g., carers, family members, or local pharmacists).
**C.**	Gary is a GP treating a patient Sam, a 19-year-old diagnosed with multiple conditions (Asthma and Diabetes), for the last ten years. Sam`s life structure has changed since he has recently become a full-time student, moved away from home, started working part-time, and engaged in a busy social life. Gary would like to learn about behavioural supports to help Sam manage taking his medication as prescribed, filling his prescription in time, and carrying multiple medications around between college, work and home. Gary also wants to find out more about solutions for cost of buying his medication each month. He also wants to learn about better ways to communicate with Sam and support his decision-making process in relation to taking his medications. To reduce Sam`s medication burden, Gary wants to increase communication and collaboration with Sam`s Pharmacist to reduce medication burden.
**D.**	GP Maria is interested in accessing information to behavioural supports to facilitate her patient Sarah, a 40-year-old female diagnosed with multiple conditions (Breast Cancer, Depression, and Chronic Insomnia), in filling her anti-depressant prescription every month. Maria wants to learn about ways to improve communication with Sarah and find strategies to help her manage side effects of treatment and medication burden. She wants to find information about the best ways to support Sarah`s decision-making.

### Analytical approach

A descriptive and exploratory approach to textual data analysis was employed, with qualitative content analysis informing the adopted analytical strategy ( Elo & Kyngäs, 2008; Elo *et al.*, 2014). An inductive approach was taken ( Hsieh & Shannon, 2005) and; given that the overall aim of the analysis was to produce an integrated picture of diverse stakeholders` perspectives on barriers, solutions to those barriers, and the user needs, a low level of data transformation was attempted and the main focus was on the analysis of manifest content and a broad surface structure ( Bengtsson, 2016; Sandelowski & Barroso, 2003). Computer assisted software was not used for this process. Qualitative analysis for the purposes of identifying categories of barriers was conducted manually by the team. The phenomena description was achieved by following relevant guidelines and the analytical process included stages of preparation, organisation, and report writing ( Elo & Kyngäs, 2008). Specifically, after immersing in the data and obtaining the sense of whole, the researchers engaged in the process of open coding and category creation using the Paired Comparison Method ( Warfield & Cardenas, 1994). Pairs of barriers were systematically assessed for conceptual similarity in turn, during an exhaustive, immersive process of comparative analysis. These conceptually similar barriers were then grouped under higher order categories. A description resulted from abstraction as well as the generation of categories and subcategories. The reporting of the analytical process has been supported by figures that represent conceptual models and illustrate the results.

### Ethical considerations

As the work described in this paper relates to a PPI expert advisory group, the project team deemed that those involved those involved should not be classified as ‘research participants’, this was more akin to an expert consensus guideline group. Such an approach aligns with the equal partnership spirit of this PPI work. Therefore the usual processes of seeking ethical approval and informed consent that pertain to health research did not apply in this work. This approach is consistent with guidance provided by the National Institute of Health Research in the UK, which classify this kind of work as an ‘ involvement activity’ and consistent with consensus statement papers that have been published by the National Research Ethics Service (NRES) and INVOLVE.

## Results

In total, 67 barriers to supporting people with multimorbidity who are taking multiple medications were identified across eight barrier categories: Training and Education; Conflict; Communication; Ownership and Responsibility; Time Pressure; Resources and Support; Patient Behaviour and Abilities; and Perspective. During the CI workshop, participants generated 162 options for overcoming barriers across the eight categories (see Tables 2–9). In the final stage of the workshop, the group focused on scenario-based specification of user needs to inform the design of an e-learning tool for health practitioners. A broad range of information and knowledge needs, communication needs, decision-making support needs, behavioural support needs, relational needs, and ‘other’ needs were identified (see Tables 10 and 11 (extended data ( Hanlon *et al.*, 2020)). A summary of some key needs is provided below. 

The *Information and Knowledge Needs* category contains the largest set of generated needs and refers to information and knowledge which would be beneficial in supporting practitioners who work with patients with multimorbidity taking multiple medications. It was suggested that the e-learning tool should provide access to information and guidance on how to address issues of adherence and non-adherence, methods that can be used to identify non-adherent patients, information on how to discuss side-effects, and details of available information resources and supports that would serve to support healthcare professionals in working with patients.

The category of *Communication Needs* highlights the need to provide prompts and guidelines for healthcare professionals in opening and structuring conversations about adherence, including sample questions which may be useful for both the healthcare professional and patient, to help them to communicate effectively.

The *Decision-making Support Needs* category includes needs relating to adherence assessment tools and methods, including methods for patients to weigh up costs and benefits associated with medications, and to generate a visualisation of this information, in addition to knowledge on how to optimise decision-making in relation to adherence. It was argued that decision-making methods that support the ability to represent side-effects on one hand and health benefits on the other hand would support more balanced reasoning and decision-making on the part of healthcare professionals and patients as they work together. 

Within the category of *Behavioural Support Needs*, participants identified the need to provide a guide on evidence-based psychological or behavioural supports and interventions, and how to implement these, such as: habit-forming supports, targeting beliefs and concerns, goal monitoring, medication reminders, action planning, and social supports which may be used by healthcare professionals to help patients with their adherence. To make such supports more engaging, it was suggested that they be presented in two forms: written information, and animations.

The *Relational Needs* category addressed needs such as promoting empathy an understanding, providing personal support and advice, and working with patients to explore key aspects of their specific circumstances and lifestyle choices, such as coping with adherence and organising their routine accordingly. For example, in the context of a patient whose living and relational arrangements have changed, understanding, empathy, support, and advice is needed to explore how patients are managing their medication in this new environment, and whether or not their daily medication routines need to be reorganised to work with this new context.

Finally, some participants also specified ‘other’ needs such as wanting more support from GPs so that they could feel less burdened and wanting more time in consultations so that they could better explain conditions and symptoms. For the full list of categorised needs that were identified see Table 11 (extended data ( Hanlon *et al.*, 2020)).

**Figure 2.**
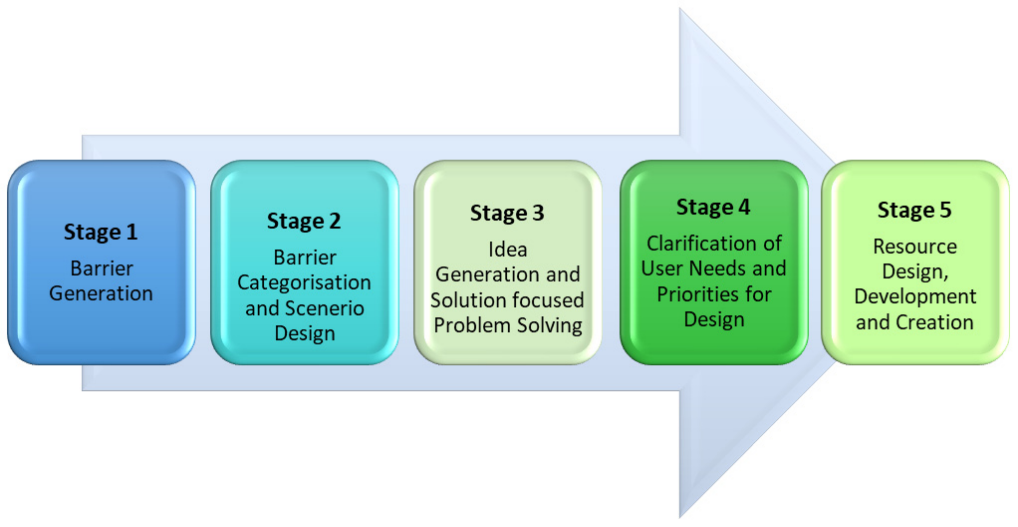
Process of resource design.

In summary, combining CI with SBD approaches, the CI session reported here was conducted to enhance understanding of complex issues with respect to (1) Barriers to supporting people with multimorbidity who are taking multiple medications; (2) Options for overcoming these barriers; and (3) Specific needs of users of an e-learning tool designed to support health practitioners caring for patients taking multiple medications. Following the session, a detailed report of the CI that was gathered from the group was generated. Further prioritisation of user needs and story-board sequencing of key learning goals was subsequently performed by the research team, which resulted in the design of the e-learning tool, which is available at www.aminuteforadherence.ie. A summary of the process can be found in Figure 2.

## Discussion

Utilising CI and SBD methodology, input was gathered from 16 stakeholders (general practitioners, practice nurses, pharmacists, medical educators, psychologists, patient and public involvement support workers as well as members of the public living with multimorbidity and receiving polypharmacy) to gain insights into barriers to supporting people with multimorbidity who are taking multiple medications, options for overcoming these barriers, and user needs relevant to the design an e-learning tool to support health practitioners caring for patients taking multiple medications. An e-learning expert on the research team provided guidance on presenting this content in the format of an e-learning resource and developing suitable assessment methods. In the short e-learning training resource that was created ( www.aminuteforadherence.ie), reasons for non-adherence are explored and advice on how to approach the topic with patients is presented. Furthermore, guidance on advising patients on simple strategies that have been shown to be effective for supporting non-adherence are provided. It is intended that the resource will be used by GPs and practice nurses that are working in a general practice setting. The e-learning training resource takes about an hour to fully complete. There are quizzes built in throughout the resource and when GPs and practice nurses complete the course, they are awarded 1 internal continuous professional development point/credit for this hour of activity by the Irish College of General Practitioners (ICGP). A suggested template for an audit that meets the requirements of the Irish Medical Council is also included on the resource website. The resource is currently being used as a teaching/training tool by the ICGP and on relevant undergraduate programmes at the National University of Ireland, Galway.

The barriers identified by the stakeholder group are numerous and diverse, which reflects the complexity of medication adherence for people living with multimorbidity in daily life. This diversity of barriers identified in our study is largely consistent with previous findings. For example, in their systematic review of systematic reviews, Kardas *et al*. (2013) identified 771 individual factors associated with treatment initiation, implementation, and persistence, reflecting socio-economic, healthcare team- and system-related, condition-related, therapy-related, and patient-related factors. Although many of these factors are relatively fixed, many more are inherently modifiable through effective clinical and behavioural intervention. Evidence suggests that physicians can be trained to better promote treatment adherence in clinical practice ( Dragomir *et al.*, 2019; Schneider *et al.*, 2004). The use of healthcare services to support health behaviour change is vital for promoting public health among our aging population. Therefore designing, testing, and implementing rigorous and feasible training programmes aimed at improving behaviour change communication skills among physicians in the context of chronic disease management is critical to ensure effective delivery of care and treatment success.

Using a CI approach that was informed by PPI principles was highly beneficial for the purposes of this work as it ensured that the insights of key stakeholders were equally represented and that the output that was produced was relevant, useful and acceptable so that research waste could be avoided ( Brett *et al.*, 2014; Chalmers *et al.*, 2014; Gray-Burrows *et al.*, 2018). Using CI methodology also ensured that the perspectives and input from the patient representatives was considered from the start (their responses to the trigger question were used to inform the design of the exercises for the workshop); that individuals with multimorbidity and taking polypharmacy were involved at all stages of the process (patient representatives took part in the same activities as healthcare professionals and researchers on the day, were also asked to provide feedback on the report that was generated following the session, and were invited to review the resource that was created to make sure that it was relevant) and; that patient representatives were given equal opportunities to express their insights (the working groups on the day all included a person living with multimorbidity and receiving polypharmacy at each table and each member of the group was given opportunities in both written and verbal form to express insights and opinions). Having the perspective of people living with multimorbidity and taking polypharmacy was thus a vital component of this work and their insights on the day of the workshop were crucial for the project. Equally, having healthcare professionals (GPs, a nurse, and a pharmacist) present ensured that the proposed strategies to overcome the identified barriers were considerate of the practical and resource demands faced in clinical settings, resulting in a resource containing strategies that are both acceptable and implementable. Finally, including health researchers with experience of applying behavioural science to complex contemporary health problems like adherence for people with multimorbidity provided important insight. Having the voices of all of these key stakeholders was vital to achieve the intended outcomes of the research project (i.e., a clinically useful tool to support healthcare professionals to promote adherence for people with multimorbidity). The use of CI methodology ensured that the group interacted effectively with each other during the session, saw problems from each other’s perspectives, and considered obstacles from a systems thinking perspective when working together to generate options for addressing adherence and content for the resource. Cumulatively, this resulted in the production of a highly relevant and comprehensive tool to address the complex clinical problem of poor adherence.

The current study must be considered in light of certain limitations. Necessitating the involvement of such a diverse group of stakeholders, though essential to produce the type of data we needed to address the aims of this work, presented certain practical challenges. Reconciling the needs and preferences of healthcare professionals, researchers, and patients in terms of dates, times and settings for the workshop was a challenge and may have excluded certain people for participating. In addition, requiring participants to commit to a full day on-site may have prevented some people from participating (e.g., people with caring responsibilities, people with diverse physical needs, etc.). Providing other means of contributing to the CI process (e.g., allowing interested parties to submit responses to the trigger question without having to attend the workshop) could ameliorate some of the barriers to participation and improve the representativeness of the data obtained.

Limitations notwithstanding, and consistent with other recent applications ( Broome, 2006; Domegan *et al.*, 2016; Hogan *et al.*, 2017), the current study highlights the value of using a scenario-based and collective intelligence approach to system design. The online resource generated from this project represents a promising tool with the potential to support primary healthcare providers to promote optimal adherence among their patients. Further research is now required to evaluate the acceptability and effectiveness of the resource as a professional training tool in routine clinical practice. How best to maximise the impact of such a resource in ever demanding professional contexts merits specific consideration. Future work utilising a similar approach to develop a resource targeting people living with multimorbidity in the community may also be warranted. This could augment the value of this resource by helping individual patients to develop medication-taking strategies that suit their unique situations, and by facilitating improved communication between patients and their healthcare providers.

## Data availability

### Underlying data

Raw Data are not publicly available as the transcripts cannot be sufficiently de-identified by redaction. Data will be made available by reasonable request to the corresponding author. A request is considered reasonable where the intended use for the data is clearly outlined, and where this intended use does not violate the protection of participants, or present any other valid ethical, privacy, or security concerns.

### Extended data

Open Science Framework: Designing an e-learning tool to support health practitioners caring for patients taking multiple medications. https://doi.org/10.17605/OSF.IO/QGYU2 ( Hanlon *et al.*, 2020)

This project contains the following extended data:

- A list of all of the statements received in response to the trigger question/problem statement sent in advance of the collective intelligence workshop
- Table 11: Full set of categorised user needs that were identified during the CI workshop

### Reporting guidelines

COREQ checklist for ‘Designing an e-learning tool to support health practitioners caring for patients taking multiple medications.’ https://doi.org/10.17605/OSF.IO/QGYU2 ( Hanlon *et al.*, 2020)

Data are available under the terms of the Creative Commons Zero "No rights reserved" data waiver (CC0 1.0 Public domain dedication).
