# Supplementary material for: Designing an e-learning tool to support health practitioners caring for patients taking multiple medications
Source: HRB Open Res. 2021 Apr 6;3:59. Originally published 2020 Sep 1. [Version 2] doi: 10.12688/hrbopenres.13110.2 (PMC8063539; doi:10.12688/hrbopenres.13110.2)
Supplement: Peer review history

# Peer review: Reviewer response for version 2

**DOI:** 10.21956/hrbopenres.14428.r29253

**Published:** 2021-04-16

**Authors:** Peter Bower

**Affiliations:** 1 NIHR School for Primary Care Research, Centre for Primary Care and Health Services Research, School of Health Science, Faculty of Biology, Medicine and Health, University of Manchester, Manchester, UK

I have now viewed the revision and I am happy to approve the revised version.

Is the work clearly and accurately presented and does it cite the current literature?

Yes

If applicable, is the statistical analysis and its interpretation appropriate?

Not applicable

Are all the source data underlying the results available to ensure full reproducibility?

Yes

Is the study design appropriate and is the work technically sound?

Yes

Are the conclusions drawn adequately supported by the results?

Yes

Are sufficient details of methods and analysis provided to allow replication by others?

Partly

Reviewer Expertise:

Health Services Research.

I confirm that I have read this submission and believe that I have an appropriate level of expertise to confirm that it is of an acceptable scientific standard.

# Peer review: Reviewer response for version 2

**DOI:** 10.21956/hrbopenres.14428.r29254

**Published:** 2021-04-07

**Authors:** Ngaire Kerse

**Affiliations:** 1 Department of General Practice and Primary Health Care, School of Population Health, Faculty of Medical and Health Sciences, University of Auckland, Auckland, New Zealand

Comments have been addressed.

Is the work clearly and accurately presented and does it cite the current literature?

Partly

If applicable, is the statistical analysis and its interpretation appropriate?

Not applicable

Are all the source data underlying the results available to ensure full reproducibility?

No

Is the study design appropriate and is the work technically sound?

Yes

Are the conclusions drawn adequately supported by the results?

Yes

Are sufficient details of methods and analysis provided to allow replication by others?

Yes

Reviewer Expertise:

Primary care, ageing, multimorbidity.

I confirm that I have read this submission and believe that I have an appropriate level of expertise to confirm that it is of an acceptable scientific standard.

# Peer review: Reviewer response for version 1

**DOI:** 10.21956/hrbopenres.14216.r28531

**Published:** 2021-02-08

**Authors:** Ngaire Kerse

**Affiliations:** 1 Department of General Practice and Primary Health Care, School of Population Health, Faculty of Medical and Health Sciences, University of Auckland, Auckland, New Zealand

Overall the paper describes a development process and is useful, but it could be shortened considerably.

1. The abstract should state who the stakeholders were.
2. The introduction is comprehensive. The aim should be clearly stated.
3. Table 2-9 could be combined into one.
4. The scenarios are only really about information finding…it is not clear what the scenarios were used for exactly.
5. It is unusual that the data is textual, was analysed and grouped, there are consumers involved, and yet consent was not necessary? 
6. The discussion goes beyond the paper in outlining what the resource is used for and repeats a lot of the methods? Perhaps an aim could encompass describing the process all the way through and the discussion could be shortened to remove the repetition.

Is the work clearly and accurately presented and does it cite the current literature?

Partly

If applicable, is the statistical analysis and its interpretation appropriate?

Not applicable

Are all the source data underlying the results available to ensure full reproducibility?

No

Is the study design appropriate and is the work technically sound?

Yes

Are the conclusions drawn adequately supported by the results?

Yes

Are sufficient details of methods and analysis provided to allow replication by others?

Yes

Reviewer Expertise:

Primary care, ageing, multimorbidity

I confirm that I have read this submission and believe that I have an appropriate level of expertise to confirm that it is of an acceptable scientific standard, however I have significant reservations, as outlined above.

# Response

**Published:** 2021-03-12

**Authors:** Michelle Hanlon

**Affiliations:** National University of Ireland, Galway, Ireland

Thank you very much for taking the time to review our paper and for providing this helpful feedback. Please find our responses to your suggestions below. 

**The abstract should state who the stakeholders were.**

 Thank you for this suggestion. We are limited with word count in the abstract but have amended it to state  ‘key stakeholders in medication adherence’. We hope that this provides a better link to the list of stakeholders, and their area of expertise, that we provided in the introduction.

**The introduction is comprehensive. The aim should be clearly stated.**

 Thank you for this helpful comment. We have amended the introduction accordingly.

**Table 2-9 could be combined into one.**

 Thank you for this suggestion. Formatting restrictions prevent us from displaying all of the tables together within one document unfortunately. The authors did not opt to combine Tables 2-9 in to one single table as we feel they are all distinct topics/areas of concern and that having space in between them makes this distinction clearer and makes them easier to read. 

**The scenarios are only really about information finding…it is not clear what the scenarios were used for exactly.**

 Yes, scenarios were presented to gather information but also to aid in orientating the group within the problem space so that they could envision such a situation occurring and what supports might be needed to solve it. We have amended this section to further clarify this.

**It is unusual that the data is textual, was analysed and grouped, there are consumers involved, and yet consent was not necessary?** 

 This is in line with INVOLVE guidelines on PPI. PPI representatives were seen as expert contributors rather than research participants. We have amended the section where this rationale is explained for clarity. 

**The discussion goes beyond the paper in outlining what the resource is used for and repeats a lot of the methods? Perhaps an aim could encompass describing the process all the way through and the discussion could be shortened to remove the repetition.**

 Thank you for this suggestion. We have shortened the discussion and tried to avoid any unnecessary repetition.

# Peer review: Reviewer response for version 1

**DOI:** 10.21956/hrbopenres.14216.r27902

**Published:** 2020-09-16

**Authors:** Peter Bower

**Affiliations:** 1 NIHR School for Primary Care Research, Centre for Primary Care and Health Services Research, School of Health Science, Faculty of Biology, Medicine and Health, University of Manchester, Manchester, UK

Thank you for the opportunity to review this interesting paper on the development of an e-learning tool in the area of patient adherence. The paper is very clearly written.

 The introduction is detailed and clear, although I felt the link to the e-learning tool could be brought in earlier, to orient the reader more effectively – which might link to some of the research presented on how change in adherence is possible. Some of this is in the discussion, and might be pulled forward? This would form a bridge between the issues of adherence, and the CI method which is the focus of the paper.

 Is there any evidence on the effectiveness of e-learning methods in these areas?

 The description of the CI method was detailed and generally clear, although there were a few areas that could have a little more detail. For example, what exactly was meant by the ‘design laws’ in the introduction? How exactly did the Paired Comparison Method work? Examples might help here – I think readers will be interested in the specifics of how this works.

 The authors talked about diversity in the sample, and that seemed true in terms of roles. What about diversity of patients in terms of SES and (possibly) ethnicity? This is often a criticism of PPI work and I wondered if it was worth a reflection here.

 The benefits of the methodology are clearly articulated, but I would have liked to see a short reflection on advantages and disadvantages compared to other methods that people might employ to generate content (such as conventional focus groups, or interviews, or less structured methods). I thought that would add to the methodological impact of the paper to have a short compare-and-contrast.

Is the work clearly and accurately presented and does it cite the current literature?

Yes

If applicable, is the statistical analysis and its interpretation appropriate?

Not applicable

Are all the source data underlying the results available to ensure full reproducibility?

Yes

Is the study design appropriate and is the work technically sound?

Yes

Are the conclusions drawn adequately supported by the results?

Yes

Are sufficient details of methods and analysis provided to allow replication by others?

Partly

Reviewer Expertise:

Health Services Research.

I confirm that I have read this submission and believe that I have an appropriate level of expertise to confirm that it is of an acceptable scientific standard, however I have significant reservations, as outlined above.

# Response

**Published:** 2021-03-12

**Authors:** Michelle Hanlon

**Affiliations:** National University of Ireland, Galway, Ireland

Thank you very much for taking the time to review our paper and for providing helpful feedback. Please find our responses to each of your comments below.

**The introduction is detailed and clear, although I felt the link to the e-learning tool could be brought in earlier, to orient the reader more effectively – which might link to some of the research presented on how change in adherence is possible. Some of this is in the discussion, and might be pulled forward? This would form a bridge between the issues of adherence, and the CI method which is the focus of the paper.**

 Thank you for this helpful suggestion. We have now made this link clearer and made reference to the e-learning tool earlier in the introduction.

**Is there any evidence on the effectiveness of e-learning methods in these areas?**

 We have now added some background on this in the introduction and provided a reference to a systematic review on the topic.

**The description of the CI method was detailed and generally clear, although there were a few areas that could have a little more detail. For example, what exactly was meant by the ‘design laws’ in the introduction? How exactly did the Paired Comparison Method work? Examples might help here – I think readers will be interested in the specifics of how this works.**

 Thank you, we have amended this part to include more detail.

**The authors talked about diversity in the sample, and that seemed true in terms of roles. What about diversity of patients in terms of SES and (possibly) ethnicity? This is often a criticism of PPI work and I wondered if it was worth a reflection here.**

 Thank you, yes we would agree that this can often be an issue. Our only criteria was that PPI representatives had multimorbidity and were receiving polypharmacy. The PPI contributors that took part were all from the same PPI panel, which could be seen as a limitation. However, there is a mix of gender, ages and backgrounds on this panel and each representative that was involved in this project brought a unique experience and perspective.

**The benefits of the methodology are clearly articulated, but I would have liked to see a short reflection on advantages and disadvantages compared to other methods that people might employ to generate content (such as conventional focus groups, or interviews, or less structured methods). I thought that would add to the methodological impact of the paper to have a short compare-and-contrast.**

 We have reflected briefly on this now in the methodology. While we agree it would be very interesting to delve in to further comparison of CI and other qualitative methods, it is something that would need/deserve further space than we would be able to dedicate to it in this particular paper. Given the length of this paper already, we believe that it is better to keep the focus on why we chose this method, the process involved in using it, and why it was beneficial and suitable for the project-which is outlined in both the method and discussion sections.
